# Long-Term Outcomes after Dialysis-Requiring Acute Kidney Injury

**DOI:** 10.1155/2014/365186

**Published:** 2014-08-12

**Authors:** Vin-Cent Wu, Chih-Chung Shiao, Chia-Hsuin Chang, Tao-Min Huang, Chun-Fu Lai, Meng-Chun Lin, Wen-Chih Chiang, Tzong-Shinn Chu, Kwan-Dun Wu, Wen-Je Ko, Cheng-Yi Wang, Shuo-Meng Wang, Likwang Chen

**Affiliations:** ^1^Division of Nephrology, Department of Internal Medicine, National Taiwan University Hospital, 7 Chung-Shan South Road, Zhong-Zheng District, Taipei 100, Taiwan; ^2^Division of Nephrology, Department of Internal Medicine, Saint Mary's Hospital, Saint Mary's Medicine, Nursing, and Management College, Luodong, Yilan 265, Taiwan; ^3^Division of Nephrology, Department of Internal Medicine, Yun-Lin Branch, National Taiwan University Hospital, Douliou City 640, Taiwan; ^4^Department of Traumatology and Surgery, National Taiwan University Hospital, 7 Chung-Shan South Road, Zhong-Zheng District, Taipei 100, Taiwan; ^5^Department of Internal Medicine, Medical Research Center, Cardinal Tien Hospital, School of Medicine, Fu Jen Catholic University, No. 362, Zhongzheng Road, Xindian District, New Taipei City 23148, Taiwan; ^6^Department of Urology, National Taiwan University Hospital, 7 Chung-Shan South Road, Zhong-Zheng District, Taipei 100, Taiwan; ^7^Institute of Population Health Sciences, National Health Research Institutes, No. 35, Keyan Road, Zhunan 350, Taiwan

## Abstract

AKI-dialysis patients had a higher incidence of long-term ESRD and mortality than the patients without AKI. The patients who recovered from dialysis were associated with a lower incidence of long-term ESRD and mortality than in the patients who still required dialysis.

## 1. Introduction

The incidence of acute kidney injury (AKI) in hospitalized patients is increasing [1] and is associated with increased in-hospital and posthospitalization resource utilization [2]. Patient survival from an episode of AKI has been improved by advances in critical care medicine and dialysis technology, and therefore an increasing number of hospitalized patients are being discharged alive after temporary AKI [3]. Patients who survive AKI have been reported to be at a greater risk for end-stage renal disease (ESRD) than patients without AKI [4, 5], and pediatric patients without preexisting kidney disease have been reported to be at a higher risk of chronic kidney disease after AKI [6]. However, the results of long-term outcomes of patients recovering from in-hospital AKI necessitating dialysis (AKI-dialysis) have been inconsistent [5, 7–9]. Although renal recovery from AKI is associated with better renal outcomes and patient survival [10], no differences in long-term survival between those with kidney function recovery after AKI and those without AKI were observed in two population-based cohorts [11, 12]. However, in postoperative patients [13–15] and geriatric patients [16], temporary worsening of kidney function has been reported with a higher long-term mortality rate compared with non-AKI patients.

The cohorts in previous reports have mostly focused on preexisting normal or near normal kidney function [9, 17–19] or all patients with chronic kidney disease (CKD) [8]. We hypothesized that hospitalized patients surviving with temporary dialysis would have poorer long-term all-cause mortality than patients without AKI in a community-based cohort of patients with and without CKD. We also compared the long-term outcomes of hospital survivors who still required dialysis. This study was conducted using 1 million beneficiaries randomly sampled from the year 2000 by the Taiwan National Health Research Institute (NHRI) and further validated by analysis of a prospectively constructed AKI database.

## 2. Patients and Methods

### 2.1. Study Population

The Taiwan National Health Insurance (NHI) program is a nationwide insurance program that covers outpatient visits, hospital admissions, prescriptions, intervention procedures, and disease profiles for over 99% of the population in Taiwan (23.12 million in 2009). It is one of the largest and most comprehensive databases in the world and has been used extensively in various studies on prescription use, diagnoses, and hospitalizations [20–22]. In cooperation with the Bureau of NHI, the National Health Research Institute (NHRI) of Taiwan randomly samples a representative database of 1,000,000 subjects from all enrollees in the NHI program using a systematic sampling method for research purposes in the form of the NHRI database (NHRID). There are no statistically significant differences in age, gender, and health-care costs between the sample group and all enrollees according to the NHRI. The NHRID contains all claims data for these individuals from January 1999 to December 2008 and offers a good opportunity to explore the outcomes of AKI-dialysis. Because the identification numbers of all subjects in the NHRID are encrypted to protect privacy, this study was exempt from full review by the Institutional Review Board.

### 2.2. Identification of Cases and Controls

The study group consisted of those aged ≥18 years with a first diagnosis of AKI-dialysis according to International Classification of Disease, 9th revision, Clinical Modification (ICD-9-CM) code and procedure codes (including the Taiwan Classification of Procedures,* supplementary tables*). A control cohort without AKI or dialysis before and during the index hospitalization was selected for comparison (non-AKI group), matched for age, sex, diabetes mellitus (DM), and mechanical ventilation (MV) support with the study group. The index hospitalization date of the controls was limited to be within the same year as that of their matched cases.

**Figure 1:**
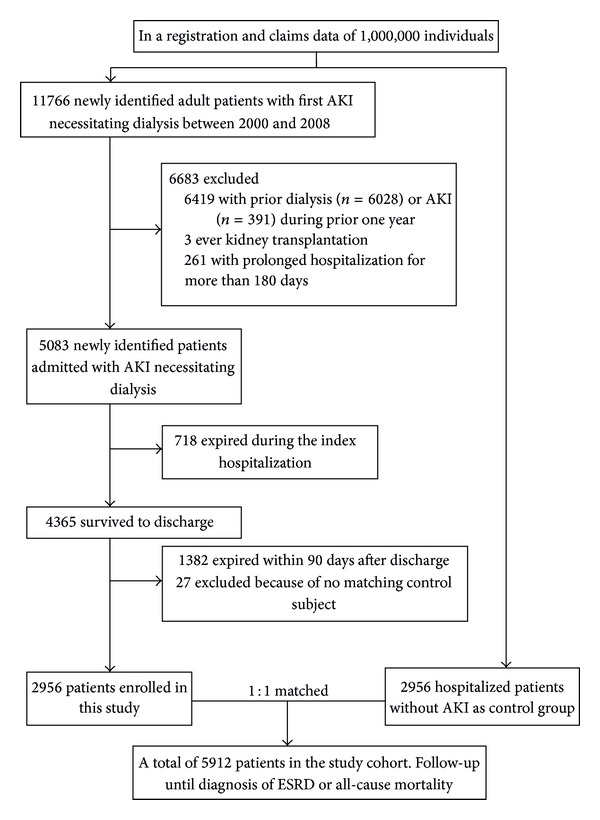
Flow diagram of the study population (AKI, acute kidney injury; DM, diabetes mellitus; ESRD, end-stage renal disease; MV, mechanical ventilation).

We used a one-year period immediately prior to the index hospitalization to identify preadmission AKI and dialysis. Patients with preadmission AKI or ESRD and those who had undergone kidney transplantation were excluded.  Figure 1 shows the patient selection flow chart. Patients with an arteriovascular fistula or implantation of a peritoneal-dialysis tube were also excluded. The AKI-dialysis patients who survived for more than 90 days after discharge from the index hospitalization and who were not readmitted to hospital were divided into two groups according to whether or not they recovered from AKI-dialysis (dialysis withdrawal and nonwithdrawal subgroups). We further defined advanced CKD as patients with a creatinine level of more than 6 mg/dl with prescriptions of concomitant erythropoiesis-stimulating agents [23]. Further, as previously reported [24], we used a selection period of 90 days to define ESRD because all patients receiving dialysis for more than 90 days in Taiwan can apply for NHI for catastrophic illness registration cards. The outcomes of this study were long-term all-cause mortality and ESRD after hospital discharge.

### 2.3. Research Variables

The demographic and clinical characteristics of the study subjects at their index hospitalization were recorded. The parameters included age, sex, year of admission, hospital characteristics, prevalence of selected comorbid conditions, Charlson comorbidity index [25], organ dysfunction developing during the index hospitalization, the categories of major operations, resource usage including hemodialysis and MV support, ICU admission, and outcomes. To determine preexisting comorbidities, we used a relatively strict criterion: at least one inpatient admission or at least three outpatient visits to treat a certain disease during the year prior to the index hospitalization. Moreover, medications including angiotensin-converting-enzyme inhibitors (ACEIs), angiotensin II receptor blockers (ARBs), statins, nonsteroidal anti-inflammatory agents (NSAIDs), diuretics, and aspirin, which are thought to influence kidney recovery [26] and were used during the 90 days after the index hospitalization, were also analyzed.

We also make an effort to examine how fluid imbalance at the initiation of dialysis could affect long-term all-cause mortality. Ideally, it would be more informative to construct a research variable to reflect the level of fluid overload based on the percentage of body weight gained. However, the NHI database does not contain data on patients' body weight. Alternatively, we constructed a proxy indicator of fluid imbalance based on the amount of medication used to control oliguria and body weight gain. We generated a variable to show whether a patient used diuretics at a level higher than 2.25 defined daily dose (DDD) at the initiation of dialysis. Each patient's exposure to diuretics (belonging to the anatomical, therapeutic, and chemical (ATC) class C03CA) was measured on the basis of the cumulative dose and expressed as the DDD according to the definition of the World Health Organization [27]. The DDD level of 2.25 was chosen as the dose equivalent of furosemide stress test (1.5 mg/kg) from a standard 60 kg patient [28]. This dosage signals a severe level of fluid imbalance.

### 2.4. Statistical Analysis

Continuous variables are described as mean ± standard deviation (SD), and discrete variables are presented as counts or percentages. All data were analyzed using R software version 2.8.1 (Free Software Foundation, Inc., Boston, MA, USA). A two-sided *P* value of less than 0.05 was considered to be statistically significant. Cox proportional hazard regression and propensity score analyses were conducted separately within each stratum to evaluate the risk of outcomes after adjustments for all variables in Table 2 and propensity score. For the outcome measurements, an individual was censored at death or at the end of the measured period.

We calculated propensity scores in an attempt to make an unbiased estimate of the confounders predicting dialysis at the 90th day after discharge, as a binary dependent variable, under a set of covariates (see Supplementary Table 1 in Supplementary Materials available online at http://dx.doi.org/10.1155/2014/365186). The presence of comorbidities was added into a nonparsimonious multivariate logistic regression model to predict dialysis at the 90th day after hospital discharge. The predicted probability derived from the logistic equation was used as the propensity score for each individual.

Due to the strong correlation between CKD, advanced CKD, ESRD, and mortality [29], we further used a Cox proportional hazards model with time-varying covariates to evaluate the impact of subsequent ESRD, advanced CKD, and CKD after discharge on the risk of mortality, assuming that changes in CKD, advanced CKD, or ESRD status could appear at a subsequent time point.

## 3. Validation

### 3.1. Propensity Matching Method for Sensitivity

The propensity score matching method was applied in the dialysis withdrawal and nonwithdrawal subgroups or non-AKI patients to reduce the effect of selection bias in the cohorts as in our previous reports [30, 31]. The subjects who did not have a suitable match within the acceptable rank range were excluded from further analysis. The models were applied to the non-AKI and nonwithdrawal groups, with the dialysis withdrawal subgroup as the reference. The patients in the dialysis withdrawal subgroup were then matched 1 : 1 separately with the nonwithdrawal subgroup and non-AKI group according to their specific propensity scores using the greedy matching technique as in our previous report [32].

### 3.2. Validation of Data Collection

The main outcome of all-cause mortality and the selection criteria to identify patients with AKI-dialysis were validated by analysis of prospectively collected data from the National Taiwan University Hospital Study Group on Acute Renal Failure (NSARF). This critical care database was constructed prospectively for outcome assessment between January 2002 and January 2008 in a single medical center (National Taiwan University Hospital in Taipei, Taiwan) and its three branch hospitals in different cities [15, 33–37] with complete information on serum creatinine (measured by following a standardized protocol). The contents of this database were used for reimbursements and are similar to those of the NHI inpatient claims files.

## 4. Results 

### 4.1. Demographic Characteristics of the Patients

#### 4.1.1. Patients with or without AKI-Dialysis

Of 5083 hospitalized patients with de novo AKI-dialysis, 718 (14.1%) died during the index hospitalization, and a total of 41.3% patients died from hospitalization to 90 days after discharge (most of whom were discharged with do not resuscitate orders [38]) (Figure 1). After excluding 27 patients for whom a matching control subject could not be found, 2956 AKI-dialysis patients were enrolled with 2956 non-AKI matched inpatients (men, 50.4%; mean age, 62.0 ± 14.8 years). In the whole cohort, the average age was 62.0 years and the Charlson score before admission was 2.62 ± 2.25. A total of 5912 patients who survived for more than 90 days after hospital discharge were included for analysis, of whom 53.8% had DM and 17.5% needed MV support (Table 1).

The patients in the AKI-dialysis group had a higher Charlson score, with more preexisting comorbidities (*P* < 0.001) and more comorbidities during the index hospitalization than the non-AKI group. More patients in the non-AKI group had a history of surgery at admission than in the AKI-dialysis group, however, and the non-AKI patients were more likely to undergo major surgery during the index hospitalization. More AKI-dialysis patients took medications including ACEIs, ARBs, statins, NSAIDs, and diuretics after hospital discharge.

#### 4.1.2. Patients with or without Withdrawal from AKI-Dialysis

Of the AKI-dialysis patients who survived to 90 days after hospital discharge, 685 (23.2%) were weaned from acute dialysis (Table 1). These patients had lower Charlson scores and a lower rate of comorbidities (myocardial infarction (MI), dementia, milder liver disease, and DM with microvascular disease) than the nonwithdrawal group. However, the dialysis withdrawal patients received more major operations, MV support, and intensive care unit (ICU) admission, with a higher rate of cardiovascular and respiratory organ failure during the index hospitalization than those in the nondialysis withdrawal group. As expected, the ratio of baseline CKD was lower in the dialysis withdrawal group than in the nonwithdrawal group.

### 4.2. Outcome Measurements

#### 4.2.1. Propensity Score Evaluation

The risk factors predicting the need for dialysis within 90 days after hospital discharge as components of propensity score are listed in Supplementary Table S1. The propensity score for predicting the need for dialysis within 90 days after hospital discharge in all study groups had a high discriminatory power (estimated area under receiver operating characteristic curves (eAUC-ROC) = 0.895) and it fitted well with the observed binary data (adjusted generalized *R* ^2^ = 0.588).

#### 4.2.2. Long-Term ESRD

**Figure 2:**
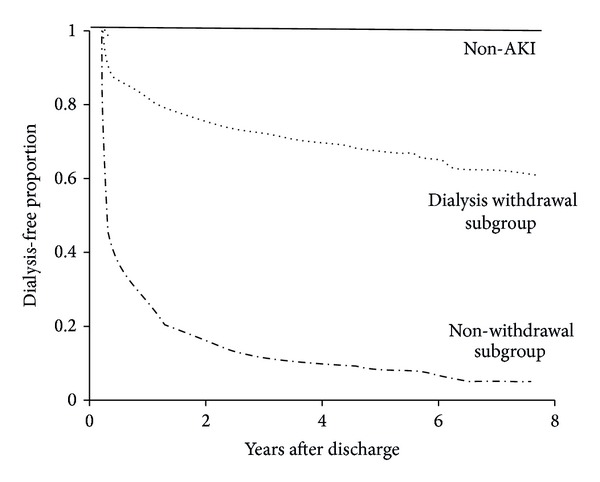
Cox proportional hazard model for long-term dialysis events of the patients alive at hospital discharge, stratified by kidney functional status after discharge (AKI, acute kidney injury).

The incidence of ESRD was 6.8 per 100 person-years among the dialysis withdrawal subgroup after a median follow-up period of 2.96 years (interquartile range (IQR)) (0.49–4.83 years). The non-AKI group had a significantly lower incidence of long-term dialysis (hazard ratio (HR), 0.05, 95% confidence interval (CI), 0.02–0.12, *P* < 0.001), and the nonwithdrawal subgroup had worse outcomes (HR, 10.38, 95% CI, 8.02–13.42, *P* < 0.001) of long-term dialysis compared with the dialysis withdrawal subgroup as the reference (Figure 2). In addition, preadmission advanced CKD (HR, 1.23, 95% CI, 1.09–1.38, *P* = 0.001), intermittent hemodialysis use during the index hospitalization (HR, 2.36, 95% CI, 1.21–4.58, *P* = 0.012), and postdischarge ACEI/ARB use (HR, 0.82, 95% CI, 0.73–0.91, *P* = 0.001) were associated with long-term dialysis.

#### 4.2.3. Long-Term All-Cause Mortality

The survivors were younger and had fewer comorbidities than the nonsurvivors (Table 2). During the index hospitalization, the survivors also had a lower rate of respiratory disease. The surviving patients received fewer cardiothoracic surgeries, used less MV support, had fewer ICU admissions, and used lower amounts of statins, aspirin, and diuretics than the nonsurvivors. Consistent with previous results, our findings showed a lower incidence of dialysis, AKI, and ESRD, but a high incidence of kidney recovery among the surviving patients (Table 2).

**Figure 3:**
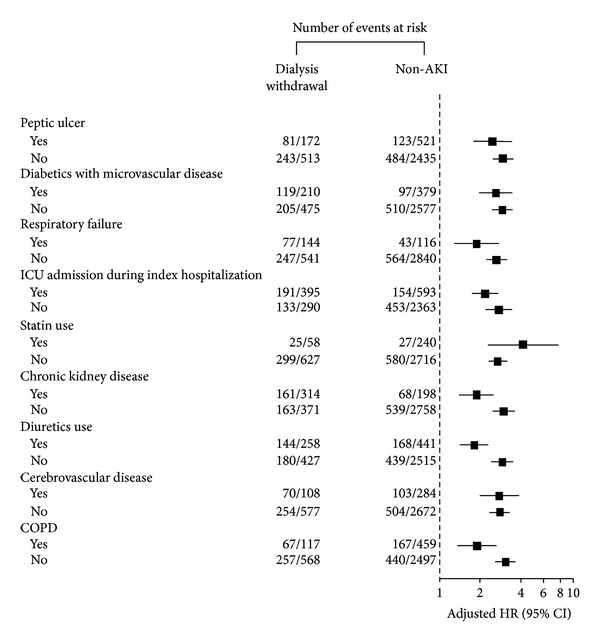
Hazard ratios and 95% confidence intervals for overall survival after adjusting for patient characteristics between the dialysis-withdrawal subgroup and the AKI-dialysis and non-AKI groups (AKI, acute kidney injury; CI, confidence interval; COPD, chronic obstructive pulmonary disease; HR, hazard ratio; ICU, intensive care unit).

Even among the dialysis withdrawal patients, the mortality rate was 14.4 per 100 person-years after a mean follow-up period of 3.29 years. The all-cause mortality rate of 4.45 per 100 person-years in the matched controls in our cohort was consistent with previous reports [5, 39]. After a mean follow-up period of 4.1 ± 2.6 years, the non-AKI group had a lower risk (HR, 0.65, 95% CI, 0.43–0.83, *P* < 0.001), whereas the nonwithdrawal subgroup had a higher risk (HR, 1.63, 95% CI, 1.39–1.92, *P* < 0.001) of long-term all-cause mortality compared with the withdrawal group. The risk was independent from use of diuretics ≥2.25 DDD at dialysis initiation (HR, 1.15, 95% CI, 1.03–1.28, *P* = 0.011) and development of subsequent ESRD (HR, 1.70, 95% CI, 1.47–1.98, *P* < 0.001) and CKD (*P* = 0.456) after discharge (Table 3). The subgroup analysis was consistent with our main finding that the non-AKI group had a survival advantage compared with the dialysis withdrawal subgroup (Figure 3).

### 4.3. Sensitivity Test

#### 4.3.1. Propensity Matching Method

After careful matching, there were 231 dialysis withdrawal patients and 231 non-AKI patients. Supplementary Table 2(a) shows the demographic data of the matched cohort. Consistent with our previous findings, the results showed that the non-AKI patients had a lower long-term mortality rate (HR, 0.62; 95% CI, 0.44–0.88; *P* = 0.007) and long-term ESRD (HR, 0.04; 95% CI, 0.01–0.14; *P* < 0.001) than the dialysis withdrawal subgroup.

We also performed one-to-one matching between the dialysis withdrawal (*n* = 543) and (*n* = 543) nonwithdrawal groups according to each patient's propensity score (Supplementary Table 2(b)). With regard to the demographic data, the nondialysis withdrawal subgroup had a higher long-term mortality rate (HR, 1.25; 95% CI, 1.06–1.48; *P* = 0.008) and ESRD (HR, 9.45; 95% CI, 6.57–13.59; *P* < 0.001) than the dialysis withdrawal subgroup.

### 4.4. Validation Using NSARF Data (Supplementary Table 3)

We validated our main findings using prospective critical care data from the NSARF. Among 234 AKI-dialysis patients, 180 (76.9%) recovered from dialysis within 90 days after discharge from index admission. In the NSARF cohort, 8788 non-dialysis patients who survival to 90 days after hospital discharge were enrolled as the controls. The baseline CKD rates, defined as patients with a baseline estimated glomerular filtration rate ≤ 60 mL/min/1.73 m^2^, were 6.9%, 30.7%, and 63.0% among the non-AKI, dialysis withdrawal, and nonwithdrawal groups, respectively. The AKI-dialysis patients had an average Charlson score of 3 ± 3.4 and an average acute physiology and chronic health evaluation II score of 18.4 ± 8.7.

Consistent with our previous findings, the results obtained using the Cox proportional hazard model showed that the nonwithdrawal subgroup had a significantly higher long-term mortality rate during the follow-up period (HR, 1.64; 95% CI, 1.05–2.56; *P* = 0.031). In addition, the non-AKI group had better survival (HR, 0.69; 95% CI, 0.54–0.87; *P* = 0.002) than the dialysis withdrawal subgroup after a median (IQR) follow-up of 3.9 years (2.38–5.65 years).

## 5. Discussion 

Patients with dialysis-requiring AKI, even temporary dialysis, had a higher long-term mortality rate than those with neither AKI nor dialysis in this large, community-based cohort of patients with and without CKD. The results using NHI data (retrospectively collected) and in NSARF data (prospectively collected) were similar. These findings are important from the perspective of a clinician caring for an individual with temporary dialysis-requiring AKI.

### 5.1. Dialysis Withdrawal and Long-Term ESRD

AKI is accompanied by extrarenal organ system failure in most patients [40]. Although there was a high mortality rate in the AKI-dialysis patients, nearly one-fifth of the surviving patients had kidney function recovery attesting to the remarkable ability of the kidneys to repair and regenerate even after severe dialysis-requiring injury. Our findings also highlight the magnitude of the problem of AKI as a cause of ESRD. According to our results, the estimated annual incidence of ESRD due to temporary dialysis was 6.8 per 100 person-years. In particular, we provided an important quantitative estimate; that is, even in survival during hospitalization and recovery of sufficient kidney function to stop dialysis, a high incidence of chronic dialysis was still required.

Severe ischemic injury results in a permanent alteration of renal capillary density, contributing to a urinary concentrating defect and a predisposition toward the development of renal fibrosis [41]. Furthermore, damage of residual kidney structure, which has been identified after AKI in animal models, includes tubular atrophy and dilation, interstitial fibrosis, and a reduction in peritubular capillary density [42, 43]. These findings suggest that AKI is associated with an increased risk of ESRD [16].

Taken together, our results show a graded relationship between AKI and ESRD, with a greater risk associated with nonrecovery from dialysis. AKI is therefore a nonnegligible cause of ESRD; however the reason why this is the case is more difficult to answer. Further research is needed to elucidate whether AKI accelerates the normal age-related decline in glomerular filtration rate [44] or whether it is a marker for other factors that are causally related to the development of kidney failure.

The impact of the initial renal replacement modality in critically ill patients with AKI on recovery of kidney function is an area of renewed interest [45]. As per our previous report, initial renal support with continuous RRT showed great advantage to dialysis independence than intermittent RRT [24].

### 5.2. Long-Term All-Cause Mortality

Our long-term outcomes do not concur with two previous studies that showed no increased mortality associated with temporary acute dialysis [11, 12]. Wald et al. reported that a prior history of AKI and dialysis was not independently associated with long-term mortality in a population-based cohort after excluding 7% of the high-risk patients [12]. The discrepancy may be attributed to the higher Charlson comorbidity score and the higher rates of DM and CKD in our study cohort from national claims data, which resulted in a higher annual mortality rate in our dialysis withdrawal subgroup (14.4% versus 10.1% in Wald et al.'s study [12]). Given the extraordinarily high rates of ESRD and mortality observed in the temporary dialysis patients, the complex interconnection between them, and the increasing incidence of both, kidney disease prevention and treatment should be a major public health priority. Furthermore, the findings of the current study also have important regional implications. Appropriate management of CKD, AKI, and ESRD is important in Taiwan not only because of the high prevalence of CKD, but also because Taiwan has the highest prevalence of ESRD in the world [46]. Consistent with our findings, use of diuretics ≥2.25 DDD at dialysis initiation predicted a worse outcome in the patients with AKI [47]. Whether the fluid imbalance was the result of more severe renal failure or whether it contributed to its cause requires further clinical trials to elucidate.

In patients with normal renal function (>90 mL/min/1.73 m^2^) prior to the renal insults who survive the precipitating cause of AKI, the overwhelming majority have been reported to recover sufficient renal function (inpatient death rate, 53%; withdrawal from dialysis, 100%) [18]. The RENAL study reported a high initial death rate (44.6%) with a low rate of those requiring maintenance dialysis (5.4% of those at day 90 after discharge). This is also supported by the fact that the enrollees in RENAL study had mean creatinine level of around 1.5–1.76 mg/dL before randomization. In patients with preexisting normal or near normal kidney function (>45 mL/min/1.73 m^2^) the inpatient death rate was 41% and withdrawal from dialysis-requiring AKI was 84% [9]. However, in patients with advanced CKD (average 15–29 mL/min/1.73 m^2^), an episode of superimposed dialysis-requiring AKI was associated with a low rate of inpatient death (28%) and a very low likelihood of recovering renal function (37.0%) [8].

Consistent with the results, the inpatient death rate of our cohort was 15.4%, and the recovery rate was low (23.2%). Thus most of the patients with dialysis-requiring AKI in this study had stage −3/4 or worse CKD, and 25.3% had advanced CKD. Therefore, most of our study group had advanced CKD superimposed with dialysis-requiring AKI, and this is consistent with our Cox analysis in the fact that baseline advanced CKD was a risk for long-term dialysis.

Accordingly, if AKI-dialysis leads to a persistent loss of renal function, then the resulting renal function impairment would account for the increased mortality [48]. Our study findings highlight that, even after adjusting for subsequent CKD or ESRD after hospital discharge, the effect of AKI is still significant. In patients after aortic surgery, the temporary worsening of renal function led to a poor long-term mortality rate compared to non-AKI patients with more preexisting comorbidities [13]. Higher comorbidities in temporary dialysis patients will result in more cardiovascular events compared with patients without AKI, and this may be the reason why AKI is a cause of mortality [49].

AKI patients with higher frailty had a higher mortality rate as expected, and the corresponding analysis would therefore lead to higher adjusted HRs for death when these patients were compared with the non-AKI patients, especially among inpatients after AKI-dialysis. This phenomenon underlies the significance of checking inherent frailty among research subjects in outcome comparisons between AKI and non-AKI. Nonetheless, the dialysis withdrawal subgroup still had poorer outcomes than those without AKI or dialysis in all scenarios. It has been reported that only 8.5% of AKI patients are referred to a nephrologist after discharge [50]. However, early postdischarge followup with a nephrologist in survivors of dialysis-requiring AKI is necessary and has been associated with a lower risk of death [51].

### 5.3. Study Limitations

Despite careful propensity score analysis, we cannot exclude the possibility of residual confounding by changes in the urine amount, serum creatinine level, and body weight gain during the index hospitalization. Nonetheless, we used procedure codes to define AKI-dialysis and focused on the patients who were weaned from temporary dialysis in the NHI reimbursement system which has a high accuracy. Further studies on dialysis-sparing AKI on patient outcomes are necessary. Second, we defined AKI as occurring from any cause and thus we were unable to detect whether the risk of progression to an adverse outcome differed among different etiologies of AKI. The major shortcoming of using administrative data as the primary basis for matching is misclassification of exposure status, specifically in this study for the presence or absence of CKD. However, the excellent performance of administrative data sets stratified by ICD-9-CM codes for AKI with dialysis has been verified to be suited for research purposes with both sensitivity and specificity of more than 90% [52].

## 6. Conclusions

Our results reinforce the view that dialysis-requiring AKI seems to independently increase all-cause mortality, even after adjusting for preexisting and subsequent CKD or ESRD. AKI itself may require specialized care to rigorously avoid potentially nephrotoxic factors, despite recovering from dialysis and AKI, after discharge that may hasten progression to long-term ESRD, and then all-cause mortality.

## Supplementary Material

The propensity model was constructed to predict dialysis at 90 days after hospital discharge. Patients in recovery or nonrecovery groups were further analyzed by propensity score matched methods. The main results of the study was further validated using a prospectively collected data from the National Taiwan University Hospital Study Group on Acute Renal Failure (NSARF).

## Figures and Tables

**Table 1:** Characteristics of patients without AKI and with AKI-dialysis.

Items	Non-AKI (n = 2956)	AKI-dialysis (n = 2956)	P	AKI-dialysis (*n* = 2956)		P^¶^	P^§^
				Nonwithdrawal subgroup (*n* = 2271)	Dialysis withdrawal subgroup (n = 685)		
Male	1490 (50.4%)	1490 (50.4%)	0.999	1097 (48.3%)	393 (57.4%)	<0.001	<0.001
Age (years)	62.0 ± 14.8	62.0 ± 14.8	0.977	61.6 ± 14.4	63.6 ± 16.0	<0.001	<0.001
Comorbidity							
Charlson score	1.6 ± 1.8	3.7 ± 2.7	<0.001	3.8 ± 2.1	3.1 ± 2.4	<0.001	<0.001
Myocardial infarction	38 (1.3%)	87 (2.9%)	<0.001	58 (2.6%)	29 (4.2%)	0.028	<0.001
Congestive heart failure	138 (4.7%)	543 (18.4%)	<0.001	411 (18.1%)	132 (19.3%)	0.499	<0.001
Peripheral vascular disease	46 (1.6%)	101 (3.4%)	<0.001	74 (3.3%)	27 (3.9%)	0.401	<0.001
Cerebrovascular disease	284 (9.6%)	469 (15.9%)	<0.001	361 (15.9%)	108 (15.8%)	1.000	<0.001
Dementia	50 (1.7%)	87 (2.9%)	0.002	53 (2.3%)	34 (5%)	<0.001	<0.001
COPD	459 (15.5%)	495 (16.7%)	0.216	378 (16.6%)	117 (17.1%)	0.815	0.323
Rheumatologic disease	31 (1.0%)	55 (1.9%)	0.012	39 (1.7%)	16 (2.3%)	0.332	0.013
Hemiplegia	24 (0.8%)	66 (2.2%)	<0.001	50 (2.2%)	16 (2.3%)	0.883	0.002
CKD	74 (2.5%)	1802 (61%)	<0.001	1595 (70.2%)	207 (30.2%)	<0.001	<0.001
Advanced CKD	3 (0.15%)	747 (25.3%)	<0.001	717 (31.6%)	30 (4.4%)	<0.001	<0.001
Solid tumor	201 (6.8%)	191 (6.5%)	0.638	137 (6%)	54 (7.9%)	0.092	0.319
Tumor with metastasis	64 (2.2%)	38 (1.3%)	0.012	20 (0.9%)	18 (2.6%)	<0.001	0.475
Diabetics with microvascular disease	379 (12.8%)	1161 (39.3%)	<0.001	951 (41.9%)	210 (30.7%)	<0.001	<0.001
Diabetes mellitus	1591 (53.8%)	1591 (53.8%)	0.999	1240 (54.6%)	351 (51.2%)	0.126	0.234
Moderate or severe liver disease	311 (10.5%)	284 (9.6%)	0.261	215 (9.5%)	69 (10.1%)	0.657	0.782
Index hospital comorbidities							
Cardiovascular	41 (1.4%)	82 (2.8%)	<0.001	29 (1.3%)	53 (7.7%)	<0.001	<0.001
Respiratory	116 (3.9%)	279 (9.4%)	<0.001	135 (5.9%)	144 (21%)	<0.001	<0.001
Hepatic	19 (0.6%)	36 (1.2%)	0.029	21 (0.9%)	15 (2.2%)	0.015	<0.001
Neurologic	11 (0.4%)	48 (1.6%)	<0.001	36 (1.6%)	12 (1.8%)	0.732	<0.001
Hematologic	14 (0.5%)	29 (1%)	0.031	26 (1.1%)	3 (0.4%)	0.122	1.000
Metabolic	1 (0%)	96 (3.2%)	<0.001	66 (2.9%)	30 (4.4%)	0.065	<0.001
Operative categories							
Cardiothoracic	98 (3.3%)	50 (1.7%)	<0.001	20 (0.9%)	30 (4.4%)	<0.001	0.169
Upper GI	33 (1.1%)	12 (0.4%)	0.002	3 (0.1%)	9 (1.3%)	<0.001	0.691
Lower GI	69 (2.3%)	20 (0.7%)	<0.001	10 (0.4%)	10 (1.5%)	0.013	0.190
Hepatobiliary	67 (2.3%)	12 (0.4%)	<0.001	4 (0.2%)	8 (1.2%)	0.002	0.073
Mechanical ventilation	516 (17.5%)	516 (17.5%)	0.999	263 (11.6%)	253 (36.9%)	<0.001	<0.001
ICU admission during index hospitalization	593 (20.1%)	933 (31.6%)	<0.001	538 (23.7%)	395 (57.7%)	<0.001	<0.001
Postdischarge medications during survey							
ACEI/ARB	576 (19.5%)	772 (26.1%)	<0.001	610 (26.9%)	162 (23.6%)	0.101	0.018
Statin	240 (8.1%)	304 (10.3%)	0.005	246 (10.8%)	58 (8.5%)	0.085	0.757
NSAID	545 (18.4%)	425 (14.4%)	<0.001	331 (14.6%)	94 (13.7%)	0.619	0.003
Aspirin	212 (7.2%)	225 (7.6%)	0.551	181 (8%)	44 (6.4%)	0.189	0.561
Diuretics	441 (14.9%)	810 (27.4%)	<0.001	552 (24.3%)	258 (37.7%)	<0.001	<0.001
Initial dialysis modality							
CVVH	—	71 (2.4%)	—	15 (0.7%)	56 (82.1%)	0.074	—
IHD	—	2885 (97.6%)	—	2256 (99.3%)	629 (91.8%)		
Use of diuretics ≥2.25 DDD at dialysis initiation	—	1446 (48.9%)	—	996 (43.9%)	450 (65.7%)	<0.001	
Outcome							
Mortality	607 (20.5%)	1371 (46.4%)	<0.001	1047 (46.1%)	324 (47.3%)	0.600	<0.001
ESRD	14 (0.5%)	1643 (55.6%)	<0.001	1520 (66.9%)	123 (17.9%)	<0.001	<0.001

^¶^Dialysis withdrawal subgroup versus nonwithdrawal subgroup; ^§^dialysis withdrawal subgroup versus non-AKI group.

(i) The data was followed up to December 2008.

(ii) The AKI-dialysis patients who survived for more than 90 days after discharge from the index hospitalization and without rehospitalization were divided into two groups according to whether or not they recovered from AKI-dialysis (dialysis withdrawal and nonwithdrawal subgroups).

(iii) Advanced CKD; patients with a creatinine level higher than 6 mg/dL with concomitant erythropoiesis-stimulating agents prescriptions.

ACEI, angiotensin-converting-enzyme inhibitor; AKI, acute kidney injury; ARBs, angiotensin II receptor blockers; CKD, chronic kidney disease; COPD, chronic obstructive pulmonary disease; CVVH, continuous venovenous hemofiltration; DDD, defined daily dose; ESRD, end-stage renal disease; GI, gastrointestinal; ICU, intensive care unit; IHD, intermittent hemodialysis; NSAIDs, nonsteroidal anti-inflammatory agents/analgesics.

**Table 2:** Characteristics of patients with or without long-term survival.

	Survival (*n* = 3934)	Nonsurvival (*n* = 1978)	*P*
Male	1946 (49.5%)	1034 (52.3%)	0.044
Age (years)	58.9 ± 14.9	68.1 ± 12.5	<0.001
Comorbidity
Charlson score	2.1 ± 2.0	3.7 ± 2.3	<0.001
Myocardial infarction	62 (1.6%)	63 (3.2%)	<0.001
Congestive heart failure	283 (7.2%)	398 (20.1%)	<0.001
Peripheral vascular disease	70 (1.8%)	77 (3.9%)	<0.001
Cerebrovascular disease	353 (9.0%)	400 (20.2%)	<0.001
Dementia	52 (1.3%)	85 (4.3%)	<0.001
COPD	497 (12.6%)	457 (23.1%)	<0.001
Rheumatologic disease	55 (1.4%)	31 (1.6%)	0.645
Hemiplegia	30 (0.8%)	60 (3.0%)	<0.001
Chronic kidney disease	1008 (25.6%)	868 (43.9%)	<0.001
Solid tumor	212 (5.4%)	180 (9.1%)	<0.001
Tumor with metastasis	38 (0.9%)	64 (3.2%)	<0.001
Diabetics with microvascular disease	819 (20.8%)	721 (36.5%)	<0.001
Diabetes mellitus	1972 (50.1%)	1210 (61.2%)	<0.001
Moderate or severe liver disease	355 (9.02%)	240 (12.1%)	<0.001
Index hospital comorbidity
Cardiovascular	73 (1.9%)	50 (2.5%)	0.100
Respiratory	200 (5.1%)	195 (9.9%)	<0.001
Hepatic	35 (0.9%)	20 (1.01%)	0.668
Neurologic	34 (0.9%)	25 (1.3%)	0.165
Hematologic	25 (0.6%)	18 (0.9%)	0.258
Metabolic	63 (1.6%)	34 (1.7%)	0.745
Initial dialysis modality			
CVVH	48 (1.2%)	23 (1.2%)	<0.001
IHD	1537 (39.1%)	1348 (68.1%)	
Use of diuretics ≥2.25 DDD at dialysis initiation	999 (25.4%)	849 (42.9%)	<0.001
Operative categories
Cardiothoracic	113 (2.9%)	35 (1.8%)	0.010
Upper GI	26 (0.7%)	19 (0.96%)	0.209
Lower GI	54 (1.4%)	35 (1.8%)	0.258
Hepatobiliary	59 (1.5%)	20 (1.01%)	0.149
Mechanical ventilation	633 (16.1%)	399 (20.2%)	<0.001
ICU admission during index hospitalization	886 (22.5%)	640 (32.4%)	<0.001
Postdischarge medications during survey periods
ACEI/ARB	875 (22.2%)	473 (23.9%)	0.149
Statin	403 (10.2%)	141 (7.1%)	<0.001
NSAID	651 (16.6%)	319 (16.1%)	0.710
Aspirin	271 (6.9%)	166 (8.4%)	0.040
Diuretics	654 (16.6%)	597 (30.2%)	<0.001
Study categories
Non-AKI	2349 (59.7%)	607 (30.7%)	<0.001
Dialysis withdrawal subgroup	361 (9.2%)	324 (16.4%)	
Nonwithdrawal subgroup	1224 (31.1%)	1047 (52.9%)	
ESRD	985 (25.0%)	672 (34.0%)	<0.001

ACEI, angiotensin-converting-enzyme inhibitor; AKI, acute kidney injury; ARBs, angiotensin II receptor blockers; COPD, chronic obstructive pulmonary disease; DDD, defined daily dose; ESRD, end-stage renal disease; GI, gastrointestinal; ICU, intensive care unit; NSAIDs, nonsteroidal anti-inflammatory agents/analgesics.

**Table 3:** Risk of long-term mortality stratified by dialysis status after index hospital discharge by Cox proportional hazard model with time-varying covariates.

	HR	Lower 95% CI	Upper 95% CI	*P*
Age (per year)	1.04	1.04	1.05	<0.001
Charlson score	1.17	1.13	1.20	<0.001
Male	1.28	1.17	1.40	<0.001
Groups				
Nonwithdrawal versus dialysis withdrawal subgroup	1.63	1.39	1.91	<0.001
Non-AKI versus dialysis withdrawal subgroup	0.65	0.43	0.83	<0.001
Dementia	1.34	1.07	1.69	0.012
Severe liver disease	1.88	1.39	2.56	0.012
During index hospitalization				
Respiratory failure during index hospitalization	1.24	1.05	1.47	<0.001
Lower GI surgery	1.63	1.15	2.29	0.005
ICU admission during index hospitalization	1.22	1.09	1.37	<0.001
Use of diuretics ≥2.25 DDD at dialysis initiation	1.15	1.03	1.28	0.011
Statin use after discharge	0.77	0.65	0.92	0.003
Diuretics use after discharge	1.28	1.15	1.41	<0.001
Time-varying ESRD event	1.70	1.47	1.98	<0.001

Adjusted generalized *R* ^2^ = 0.279; concordance index = 0.83.

AKI, acute kidney injury; CI, confidence interval; DDD, defined daily dose; ESRD, end-stage renal disease; GI, gastrointestinal; HR, hazard ratio.

## Abbreviations

ACEI: Angiotensin-converting-enzyme inhibitor
AKI: Acute kidney injury
ARBs: Angiotensin II receptor blockers
COPD: Chronic obstructive pulmonary disease
ESRD: End-stage renal disease
GI: Gastrointestinal
ICU: Intensive care unit
NSAIDs: Nonsteroidal anti-inflammatory agents/analgesics.
