# Supplementary material for: Long-Term Outcomes after Dialysis-Requiring Acute Kidney Injury
Source: Biomed Res Int. 2014 Aug 12;2014:365186. doi: 10.1155/2014/365186 (PMC4145550; doi:10.1155/2014/365186)
Supplement: Supplementary file 1 — The propensity model was constructed to predict dialysis at 90 days after hospital discharge. Patients in recovery or nonrecovery groups were further analyzed by propensity score matched methods. The main results of the study was further validated using a prospectively collected data from the National Taiwan University Hospital Study Group on Acute Renal Failure (NSARF). [file 365186.f1.pdf]

Supplementary File

Table S1. Demographics and comorbidities added into a non-parsimonious propensity model to predict dialysis at 90 days after hospital discharge.

	HR	lower 95% CI	upper 95% CI	P
**Age (per year)**	0.99	0.98	0.99	<0.001
**Baseline comorbidities**				
**Chronic kidney disease**	27.38	23.32	32.15	<0.001
**Diabetics mellitus**	0.47	0.38	0.59	<0.001
**Diabetics with microvascular disease**	1.43	1.19	1.72	<0.001
**Metabolic disease**	3.04	1.73	5.32	<0.001
**Mechanical ventilation**	0.61	0.46	0.82	0.001
**Tumor with metastasis**	0.40	0.21	0.76	0.005
**COPD**	0.80	0.64	0.99	0.037
**Operative categories**				
**Hepatobiliary**	0.21	0.07	0.65	0.007
**Cardiothoracic**	0.38	0.20	0.70	0.002
**Upper GI**	0.29	0.08	1.11	0.071
**Index hospital comorbidities**				

**Hematologic**	2.54	1.07	6.04	0.035
**Post discharge medication during survey**				
**NSAID**	0.77	0.63	0.95	0.015
**ACEI/ARB**	1.46	1.21	1.77	<0.001
**Diuretics**	0.81	0.67	0.99	0.037

Adjusted generalized R^2 =0.588,

Abbreviations,

ACEI, angiotensin-converting-enzyme inhibitor; AKI, acute kidney injury; ARB, Angiotensin II receptor blockers; COPD, chronic obstructive pulmonary disease; ESRD, end stage renal disease; GI, Gastrointestinal; NSAID, nonsteroidal anti-inflammatory agents/analgesics.

Table S2a Propensity matched of baseline characteristics and clinical endpoints between patient without AKI or dialysis and recovery from AKI-dialysis.

	**Non-AKI (n=231)**	**Recoverable subgroup (n=231)**	**p**
**Male**	133(57.6%)	126(54.5%)	0.574
**Age (years)**	62.6±15.3	63.1±15.8	0.531
Comorbidities			
**Charlson score**	2.81±2.42	3±2.33	0.256
**Myocardial infarction**	9(3.9%)	8(3.5%)	0.999

**Congestive heart failure**	38(16.5%)	36(15.6%)	0.899
**Peripheral vascular disease**	10(4.3%)	8(3.5%)	0.811
**Cerebrovascular disease**	38(16.5%)	40(17.3%)	0.901
**Dementia**	9(3.9%)	12(5.2%)	0.656
**COPD**	47(20.3%)	44(19%)	0.815
**Rheumatologic disease**	6(2.6%)	6(2.6%)	0.999
**Hemiplegia**	5(2.2%)	3(1.3%)	0.724
**Solid tumor**	11(4.8%)	15(6.5%)	0.546
**Tumor with metastatic**	4(1.7%)	5(2.2%)	0.999
**AIDS**			
**Diabetes Mellitus**	126(54.5%)	120(51.9%)	0.641
**Diabetics with microvascular disease**	57(24.7%)	68(29.4%)	0.295
**Liver disease**			
**Moderate or Severe liver disease**	21(9.1%)	20(8.7%)	0.999
**Chronic Kidney disease**	104(45%)	107(46.3%)	0.852
Index hospital comorbidity			
**Cardiovascular**	16(6.9%)	15(6.5%)	0.999
**Respiratory**	41(17.7%)	42(18.2%)	0.999
**Renal**	2(0.9%)	2(0.9%)	0.999
**Hepatic**	3(1.3%)	1(0.4%)	0.623
**Neurologic**	5(2.2%)	4(1.7%)	0.999
**Hematologic**	0(0%)	1(0.4%)	0.999

**Metabolic**	1(0.4%)	1(0.4%)	0.999
Operative categories			
**Cardio-thorathic**	16(6.9%)	12(5.2%)	0.559
**Upper GI**	2(0.9%)	1(0.4%)	0.999
**Lower GI**	3(1.3%)	4(1.7%)	0.999
**Hepatobiliary**	2(0.9%)	1(0.4%)	0.999
**Mechanical ventilation**	19(8.2%)	18(7.8%)	0.999
**ICU admission during index hospitalization**	120(51.9%)	116(50.2%)	0.780
Post discharge medications during survey			
**ACEI/ARB**	45(19.5%)	42(18.2%)	0.812
**Statin**	16(6.9%)	21(9.1%)	0.493
**NSAID**	37(16%)	30(13%)	0.428
**Aspirin**	14(6.1%)	18(7.8%)	0.583
**Diuretics**	78(33.8%)	74(32%)	0.767

ACEI, angiotensin-converting-enzyme inhibitor; AKI, acute kidney injury; ARB, Angiotensin II receptor blockers; COPD, chronic obstructive pulmonary disease; ESRD, end stage renal disease; GI, Gastrointestinal; ICU, intensive care unit; MACE, major adverse cardiovascular events; NSAID, nonsteroidal anti-inflammatory agents/analgesics.

Table S2b Propensity matched of baseline characteristics and clinical endpoints between patient with or without recovery from AKI-dialysis.

	***Non recoverable subgroup* (n=543)**	***Recoverable subgroup* (n=543)**	**p**
**Male**	286(52.7%)	298(54.9%)	0.503
**Age (years)**	62.7±15.5	63.6±15.8	0.314
Comorbidity			
**Charlson score**	3.4±2.42	3.37±2.42	0.749
**Myocardial infarction**	15(2.8%)	20(3.7%)	0.492
**Congestive heart failure**	107(19.7%)	107(19.7%)	0.999
**Peripheral vascular disease**	24(4.4%)	22(4.1%)	0.880
**Cerebrovascular disease**	84(15.5%)	97(17.9%)	0.329
**Dementia**	19(3.5%)	25(4.6%)	0.442
**COPD**	112(20.6%)	98(18%)	0.318
**Rheumatologic disease**	11(2%)	13(2.4%)	0.837
**Hemiplegia**	12(2.2%)	15(2.8%)	0.697
**Solid tumor**	36(6.6%)	40(7.4%)	0.721
**Tumor with metastatic**	13(2.4%)	12(2.2%)	0.999
**Diabetic Mellitus**	289(53.2%)	289(53.2%)	0.999
**Diabetics with microvascular disease**	195(35.9%)	184(33.9%)	0.524
**Liver disease**			

**Moderate or Severe liver disease**	56(10.3%)	57(10.5%)	0.999
**Chronic Kidney disease**	313(57.6%)	304(56%)	0.624
Index hospital comorbidity			
**Cardiovascular**	22(4.1%)	24(4.4%)	0.880
**Respiratory**	74(13.6%)	94(17.3%)	0.111
**Renal**	290(53.4%)	292(53.8%)	0.952
**Hepatic**	11(2%)	10(1.8%)	0.999
**Neurologic**	9(1.7%)	10(1.8%)	0.999
**Hematologic**	3(0.6%)	3(0.6%)	0.999
**Metabolic**	23(4.2%)	24(4.4%)	0.999
Operative categories			
**Cardiothoracic**	13(2.4%)	9(1.7%)	0.519
**Upper GI**	2(0.4%)	4(0.7%)	0.687
**Lower GI**	3(0.6%)	8(1.5%)	0.224
**Hepatobiliary**	2(0.4%)	4(0.7%)	0.687
**Mechanical ventilation**	41(7.6%)	39(7.2%)	0.908
**ICU admission during index hospitalization**	281(51.7%)	264(48.6%)	0.336
Post discharge medications			
**ACEI/ARB**	149(27.4%)	140(25.8%)	0.583
**Statin**	59(10.9%)	50(9.2%)	0.419
**NSAID**	90(16.6%)	82(15.1%)	0.561

**Aspirin**	42(7.7%)	39(7.2%)	0.817
**Diuretics**	205(37.8%)	192(35.4%)	0.450

ACEI, angiotensin-converting-enzyme inhibitor; AKI, acute kidney injury; ARB, Angiotensin II receptor blockers; COPD, chronic obstructive pulmonary disease; ESRD, end stage renal disease; GI, Gastrointestinal; ICU, intensive care unit; MACE, major adverse cardiovascular events; NSAID, nonsteroidal anti-inflammatory agents/analgesics.

Table S3

Risk of long-term mortality using the critical NSARF database by Cox proportional hazard model. (Abbreviations; AKI, acute kidney injury; DM, diabetics mellitus; CI, confidence interval; CKD, chronic kidney disease; CHF, congestive heart failure; HR, hazard ratio)

	HR	lower 95% CI	upper 95% CI	P
**Age (per year)**	1.03	1.03	1.03	<0.001
**Sex**	1.30	1.19	1.41	<0.001
**Charlson**	1.13	1.12	1.14	<0.001
**CKD**	1.47	1.23	1.75	<0.001
**Non-AKI vs. Dialysis withdrawal subgroup**	0.69	0.54	0.87	0.002
**Non- withdrawal vs. Dialysis withdrawal subgroup**	1.64	1.05	2.56	0.031
**HTN**	0.74	0.67	0.80	<0.001
**Moderate Severe liver disease**	1.14	0.85	1.52	0.377
**Mild liver disease**	1.13	0.86	1.49	0.381
**CHF**	1.48	1.31	1.68	<0.001
**DM**	1.11	1.01	1.22	0.031

Adjusted generalized R^2 =0.211, C- index= 0.76
